# A temperature-dependent virus-binding assay reveals the presence of neutralizing antibodies in human cytomegalovirus gB vaccine recipients’ sera

**DOI:** 10.1099/jgv.0.001860

**Published:** 2023-06-13

**Authors:** Ariane C. Gomes, Ilona A. Baraniak, Megan R. McIntosh, Isabella Sodi, Toby Langstone, Saima Siddiqui, Claire Atkinson, Gary R. McLean, Paul D. Griffiths, Matthew B. Reeves

**Affiliations:** ^1^​ Institute of Immunity and Transplantation, Division of Infection and Immunity, UCL, Royal Free Campus, London, NW3 2PP, UK; ^2^​ London Metropolitan University, School of Human Sciences, London, N7 8DB, UK; ^3^​ Imperial College London, National Heart and Lung Institute, London, W2 1PG, UK

**Keywords:** glycoprotein B, vaccine, cytomegalovirus, neutralizing antibodies

## Abstract

Human cytomegalovirus (HCMV) remains an important cause of mortality in immune-compromised transplant patients and following congenital infection. Such is the burden, an effective vaccine strategy is considered to be of the highest priority. The most successful vaccines to date have focused on generating immune responses against glycoprotein B (gB) – a protein essential for HCMV fusion and entry. We have previously reported that an important component of the humoral immune response elicited by gB/MF59 vaccination of patients awaiting transplant is the induction of non-neutralizing antibodies that target cell-associated virus with little evidence of concomitant classical neutralizing antibodies. Here we report that a modified neutralization assay that promotes prolonged binding of HCMV to the cell surface reveals the presence of neutralizing antibodies in sera taken from gB-vaccinated patients that cannot be detected using standard assays. We go on to show that this is not a general feature of gB-neutralizing antibodies, suggesting that specific antibody responses induced by vaccination could be important. Although we can find no evidence that these neutralizing antibody responses are a correlate of protection *in vivo* in transplant recipients their identification demonstrates the utility of the approach in identifying these responses. We hypothesize that further characterization has the potential to aid the identification of functions within gB that are important during the entry process and could potentially improve future vaccine strategies directed against gB if they prove to be effective against HCMV at higher concentrations.

## Introduction

Human cytomegalovirus (HCMV) [1] remains a major clinical burden in the population. The problem is exacerbated by the manifold threat posed by primary infection and reinfection, as well as reactivation of latent infections in the host [2]. Indeed, HCMV remains the most common viral cause of pathogenesis in the congenital infection setting and is responsible for substantial morbidity in immune-compromised hosts [2, 3]. This burden led the US National Institute of Medicine to designate HCMV the highest priority status for the development of a vaccine [4]. Despite substantial progress by a number of research groups, no vaccines against HCMV have been licensed.

The most successful vaccination strategy against HCMV to date is based on the viral fusion protein glycoprotein B (gB) [5, 6], which was administered with an MF59 adjuvant [7–9]. The natural host immune response against gB is prodigious and thus gB is considered to be highly immunogenic. A number of elegant studies have identified and characterized five antigenic domains (ADs) that predominate in the humoral response to gB in naturally infected individuals [10–16]. A proportion of these antibody responses against ADs of gB are neutralizing (nAb), although this is highly variable between HCMV-seropositive individuals and complex to measure because virion proteins other than gB are also important targets for neutralizing antibody responses. Indeed, the overall contribution of neutralizing antibodies directed against gB to control of HCMV is an area of debate: neutralizing activity associated with sera from HCMV-seropositive individuals is argued to involve a significant proportion of antibodies that recognize components of additional glycoprotein complexes present in the virion envelope (e.g. the pentameric and trimeric complexes containing gH and gL) [17–22]. Furthermore, it has been hypothesized that aspects of the humoral immune response against gB may actually be detrimental through direct competition for binding epitopes with responses considered to be protective [23]. In addition to a prodigious humoral response against gB, there is also a strong T cell response against this antigen, with responses detected against peptides that span the breadth of the gB protein [24, 25]. It is this broad immunogenicity coupled with the pivotal role that gB plays in viral infection that has supported the inclusion of gB in multiple HCMV vaccine preparations [26].

Our laboratory is interested in identifying correlates of protection associated with the gB/MF59 vaccine [7, 8, 27] – with the ultimate goal of providing mechanistic correlates of protection to inform future vaccine strategies. We have previously reported on our analyses of sera taken from HCMV-seronegative individuals subsequently vaccinated with the gB/MF59 vaccine whilst on the waiting list for solid organ transplant. In these analyses we could not detect evidence of anti-gB antibodies with neutralizing activity using standard assays prior to organ transplant [28]. This was despite clear evidence of a robust humoral response to gB overall in these vaccine recipients – with the magnitude of the response directly correlating with reduced CMV viraemia post-transplant [7]. An inability to detect evidence of a robust neutralizing antibody response was surprising but was independently observed in a separate study of sera obtained from a different gB/MF59 vaccine cohort that addressed the ability of the gB vaccine to reduce HCMV acquisition in women of child-bearing age [29]. Furthermore, the response to the gB vaccine in HCMV-seronegative and HCMV-seropositive individuals is likely to be different. Initial analyses of the sera from HCMV-seropositive individuals suggested that the gB vaccine largely boosted pre-existing immune responses against specific epitopes within gB identified in studies of natural infection, whereas, intriguingly, the response in HCMV-seronegative gB vaccinees revealed that the humoral response was distinct from that seen in response to natural infection [28, 30, 31]. Most recently, we have identified that one such vaccine-specific response was directed against epitopes overlapping within domain V of gB – a response we have called AD6 [32].

In direct contrast to our ability to detect nAbs in the sera of taken *pre-transplant*, we noted that we could identify clear evidence of nAbs directed against gB in a number of patients within the same HCMV-seronegative vaccine cohort when sera were taken and analysed early *post-transplant* [33]. Two immediate interpretations were potentially valid for these observations: (1) even though immune-suppressed, patients could rapidly generate novel gB antibody responses post-transplant, including neutralizing antibodies, or (2) HCMV-seronegative vaccine recipients did generate low level nAb responses and these were enhanced upon infection with HCMV following the transplant of an organ from a seropositive donor – essentially a two-step prime–boost event elicited by vaccine and subsequent infection post-transplant with an organ from a seropositive donor.

To investigate the hypothesis that patient sera taken pre-transplant from HCMV-seronegative individuals who were receiving gB/MF59 vaccine contained low levels of anti-gB neutralizing antibodies we employed a cold neutralization assay that alters the kinetics of HCMV infection at the plasma membrane by allowing virus to bind but not enter the cell, while potentially promoting conformational changes in gB required for fusion and entry. Using this approach, we detected evidence of neutralizing antibodies being present in the sera of HCMV-seronegative individuals who have been vaccinated with the gB/MF59 vaccine *prior to transplant*. Using a panel of HCMV gB antibodies we demonstrated that this is not a general feature of the assay and, in some cases, nAbs from standard assays actually display reduced activity in the modified assay, suggesting that the protocol does not just ‘improve’ all neutralizing antibody responses against HCMV non-specifically.

Since some of the vaccine recipients proceeded to transplant, we could investigate whether this novel activity correlated with any clinical outcomes post-transplant. However, we could find no evidence that the detection of neutralizing antibodies in pre-transplant sera was a statistically significant correlate of protection when tested against a number of clinical parameters. Although in this case no clinical correlation was observed in these limited samples available from the clinical study, they do suggest the presence of nAbs that recognize gB in certain states during the binding and fusion activity of gB during entry and that these were induced by gB vaccination. Characterization of these responses that neutralize infection in these assays has the potential to provide new insights into regions of gB that are important for the function and entry of HCMV.

## Methods

### Ethical statement

The study was approved by the UCL Research Ethics Committee and all patients whose samples were investigated here gave written informed consent [7].

### Patient population

The population from whom samples have been evaluated and described in this work comprises the highest risk cohort of seronegative solid organ transplant patients who were enrolled in a phase II randomized and double-blinded placebo controlled cytomegalovirus glycoprotein B vaccine with MF59 adjuvant trial [7]. For post-transplant analyses, HCMV-seronegative patients who received an organ from seropositive individuals were selected and have been described elsewhere [33]. In the original study this cohort represents 11 vaccine recipients and 5 placebo recipients, but for this study removal of consent for future studies and a patient death mean that the data presented are from the remaining nine vaccine and four placebo recipients. No other selection criteria were applied.

In short, the vaccine or placebo was given in three doses: at day 0 (baseline), 1 month later and 6 months later. Following vaccination, blood samples from patients were obtained consecutively. The patients who subsequently underwent transplantation were followed up and tested by real-time quantitative PCR (rtqPCR) for cytomegalovirus DNA [7]. CMV PCR was performed on a routine basis with an in-house TaqMan (ABI)-based method as described in detail by Atabani *et al*. [34]. HCMV viraemia was defined as one or more positive HCMV PCR results (cut-off, 200 genomes ml^−1^ of whole blood, equivalent to 168 IU ml^−1^). If viraemia >3000 genomes ml^−1^ was detected (equivalent to 2520 IU ml^−1^), the patient was treated with antiviral drugs as described in [7]. Exclusion criteria included: pregnancy (a negative pregnancy test was required before each vaccine dose); receipt of blood products (except albumin) in the previous 3 months; and simultaneous multi-organ transplantation [7] .

### Cells, virus and media

Human foreskin fibroblasts (HFFs) were routinely cultured in Dulbecco's Modified Eagle's Medium (DMEM) supplemented with foetal bovine serum (10%) plus penicillin and streptomycin (100 ug ml^−1^). For neutralization assays, cells were harvested by tryspinization and seeded at 80 % confluence 1 day prior to infection in 96-well plates.

The HCMV strain Merlin was propagated in HFFs and purified by density-dependent centrifugation. Merlin was used at a multiplicity of infection (m.o.i.) of 1–5 in an experiment-dependent manner.

### Neutralization assays

To assess sera for neutralizing capacity by standard assay, HCMV was pre-incubated with heat-inactivated sera for 1 h and then used to infect HFFs. Alternatively, virus was incubated with anti-gB antibodies HCMV37 (abcam) or QG1 IgG and IgA monoclonal antibodies targeting AD-2 of gB that have been characterized previously [35]. After 24 h, cells were fixed and stained for IE gene expression using anti-IE (Millipore; 1 : 2000) and goat anti-mouse Alexa Fluor 568 nm (Life Technologies; 1 : 1000). Nuclei were counterstained with DAPI (Sigma). Percentage infection was enumerated using Hermes WiScan instruments and software.

To perform the modified neutralization assay, HFFs were infected at +4 °C for 1 h with HCMV. Then antibodies or sera were added directly to the target well and incubated at +4 °C for an additional 1 h. Cells were then shifted to 37 °C to promote virus internalization and washed after 2 h with phosphate-buffered saline (PBS), and infection was scored as described for a standard assay (above).

For experiments testing the effect of complement, 5 % guinea pig complement (Sigma) was added to the heat-inactivated sera at the time of incubation of sera with the virus–cell infections.

## Results

**Fig. 1.**
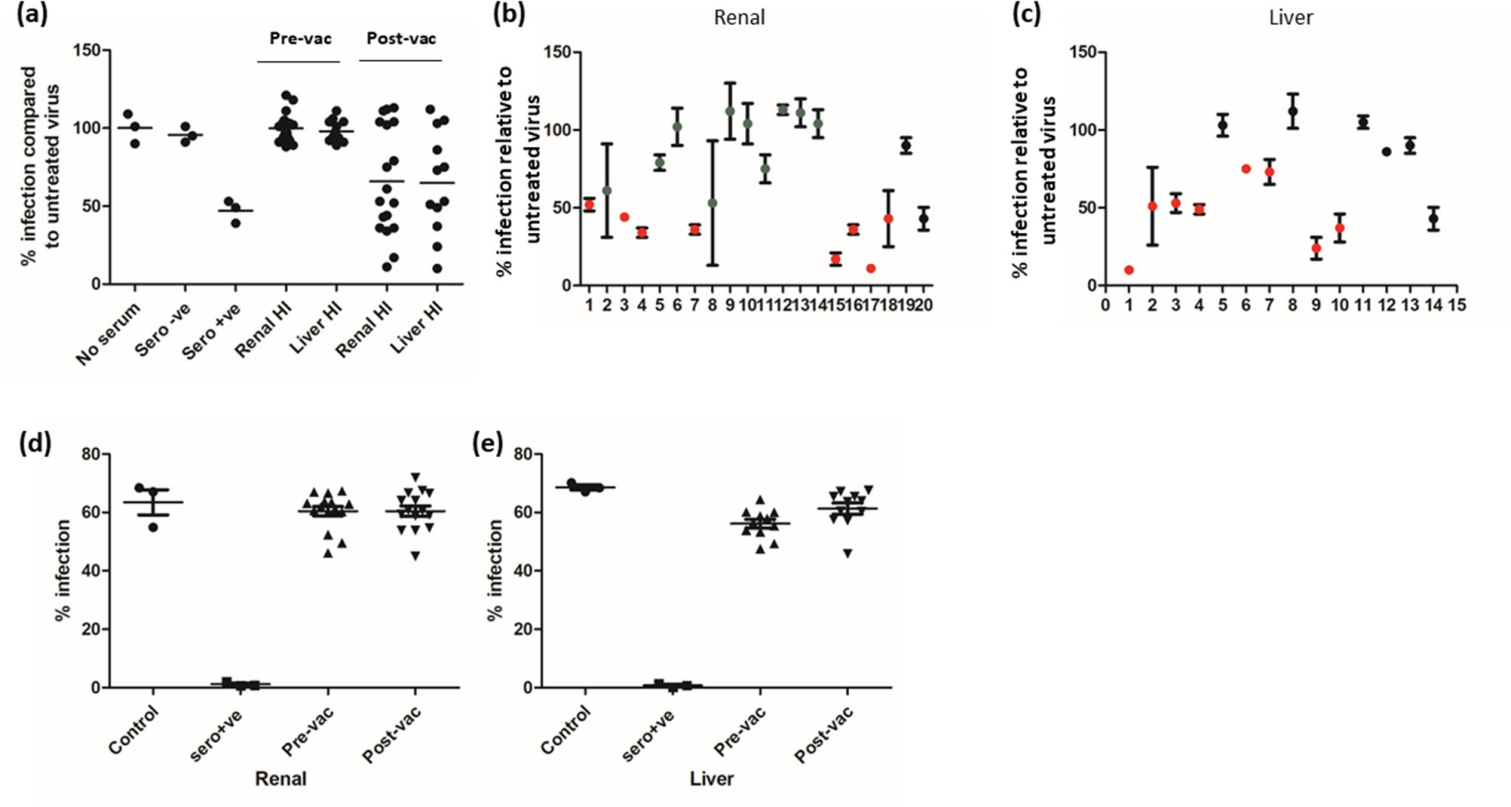
Detection of low levels of neutralizing antibodies in gB vaccine sera pre-transplant. (a–e) Sera from 30 HCMV-seronegative transplant recipients at time of transplant were heat-inactivated (HI) and then either incubated with cells pre-infected with HCMV at +4 °C [(a–c); (a) all samples and (b, c) the same analyses but separated into renal (18) and liver (12) transplant cohorts] or with HCMV prior to infection (**d, e**). Cells were then incubated at 37 °C and analysed for IE gene expression by indirect IF staining 24 h later. Infection was quantified by Hermes wiscan automated counting. For studies of human sera each dot represents the mean of three independent experiments performed in duplicate on individual patient sera. In (b, c) a red dot denotes patients with sera that significantly (*P*<0.05) neutralized infection at +4 °C compared to the same sera analysed at 37 °C (**d, e**) in a standard assay. Also in (b, c) control HCMV-seronegative sera (lanes 19 and 13, respectively) and control HCMV-seropositive sera (lanes 20 and 14, respectively) are shown.

Routinely, we interrogate sera for the presence of neutralizing antibodies using a standard assay that incubates cell-free HCMV with sera for 1 h prior to infection of permissive cells. Using this approach we could find little evidence for neutralizing antibody activity in the sera of a cohort of individuals who were HCMV-seronegative prior to gB/MF59 vaccination [7, 28]. However, in an unrelated study of an inhibitor of viral entry (DIDS) we noted that pre-absorption of HCMV virions to the cell at +4 °C dramatically changed the antiviral profile of DIDS [36] and thus we decided to test what impact pre-absorption of the virus had on neutralizing antibody activity against HCMV. To do this, HFFs were first inoculated with HCMV at +4 °C for 1 h and then incubated with sera from seronegative vaccine recipients either pre-vaccine (day 0) or post-vaccine (day of transplant) for a further 1 h, also at +4 °C. The tissue culture plates were then shifted to 37 °C to trigger viral entry and infection scored by immunostaining for IE-positive cells 24 h post-infection (p.i.) (Fig. 1). As expected, the sera taken pre-vaccine had no impact on virus infection but, interestingly, the data show clear evidence of virus neutralization under these experimental conditions in some but not all patient sera taken post-vaccination (Fig. 1a). A more detailed analysis of each individual serum sample taken from renal (Fig. 1b) and liver (Fig. 1c) transplant candidates revealed that the neutralization reached significance compared to seronegative control sera. In contrast, and consistent with a previous report [28], these same sera did not neutralize HCMV infection in a standard assay in both pre-and post-gB vaccination samples (Fig. 1d, e), suggesting that the +4 °C assay could be revealing potential neutralizing antibody responses.

Before investigating this further we decided to test whether the ability to detect evidence of neutralizing antibody activity in our vaccine recipients’ pre-transplant sera using this modified assay could simply reflect an artefact of an *in vitro* experimental approach that amplifies the activity of low-level neutralizing antibody responses against gB. Thus we tested whether this approach enhanced the ability of known gB antibodies when they were used at concentrations that elicited partial neutralization of viral infection.

**Fig. 2.**
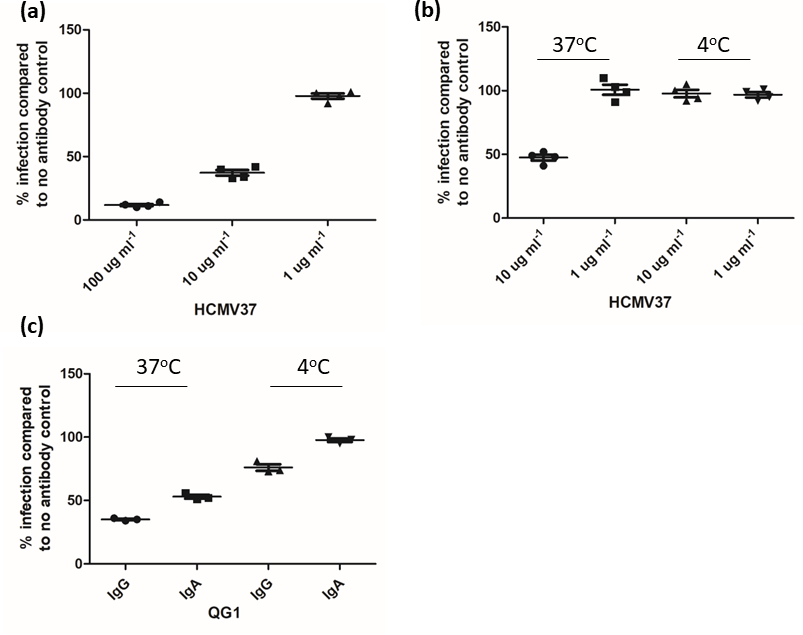
Neutralizing activity of known gB nAbs is not enhanced at +4 °C. (a) HCMV was incubated with HCMV37 antibody at 100, 10 and 1 ug ml^−1^ for 1 h prior to infection and scored for neutralizing activity by indirect IF for IE protein expression. (b, c) HCMV was incubated with HCMV37 (**b**) or QG1 IgG or IgA (**c**) prior to infection (Pre) or HFFs were infected at +4 °C for 1 h and then incubated for a 1 h with HCMV37 (**b**) or QG1 IgG or IgA (**c**) before shifting to 37 °C to promote infection (Post). At 24 h p.i., infection was scored by indirect IF for IE protein expression. The mean of four independent experiments performed in duplicate is shown.

Our first analyses focused on the gB antibody HCMV37 [37]. First we identified a concentration at which HCMV37 partially neutralized HCMV infection under our standard assay conditions (Fig. 2a). Next, we tested whether HCMV37 was able to neutralize HCMV under our modified conditions. To do this, cells were incubated with HCMV for 1 h at +4 °C and then incubated with HCMV37 for a further hour at +4 °C before shifting to 37 °C to promote internalization. As expected, HCMV37 (10 ug ml^−1^) partially neutralized HCMV that was lost at a higher dilution in a standard assay (1 ug ml^−1^; Fig. 2b). However, neutralizing activity associated with HCMV37 was completely lost in our modified assay (Fig. 2b), opposite to our observations with vaccine patient sera (Fig. 1).

To investigate whether this was HCMV37-specific, we took advantage of two monoclonal antibodies directed against AD2 that we have previously reported to neutralize HCMV infection [35]. Using the same experimental approach, we incubated HCMV with QG1 IgG and IgA monoclonal antibodies directed against the AD2 epitope as before at concentrations known to promote partial neutralization. Similar to the data with HCMV37, we observed that the neutralizing activity of both the IgG and IgA of QG1 was more efficient if pre-incubated with HCMV virions in a standard neutralization assay. Specifically, for both antibodies the neutralizing activity was again lower when added to cells post-virus absorption at +4 °C, although, unlike with HCMV37, neutralizing activity was not completely abolished (Fig. 2c). Thus whilst the pre-binding of HCMV to the plasma membrane at +4 °C was potentially revealing neutralizing antibody activity in our vaccine sera it was not enhancing the activity of all the gB neutralizing antibodies used routinely in the laboratory.

**Fig. 3.**
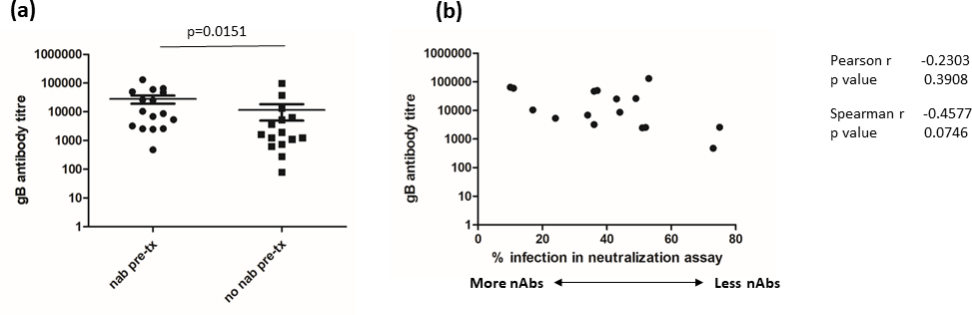
Total gB response does not completely predict those with neutralizing antibodies. (a) The total gB antibody titre in patient sera was calculated for pre-transplant sera from 31 HCMV-seronegative vaccine recipients that had been defined as possessing neutralizing (nAb pre-tx) or no neutralizing (no nAb pre-tx) antibodies. A comparison of the means was performed using Mann–Whitney U test assuming a non-parametric distribution. (**b**) Sera from patients identified as possessing neutralizing activity were then analysed by linear regression comparing neutralizing activity versus antibody gB antibody titre by Pearson and Spearman rank analysis.

To understand the relative importance of the detection of antibodies with neutralizing activity in our vaccine-recipient sera under these modified experimental conditions we investigated whether one explanation for the observed neutralization in some patients’ sera was that individuals who made large overall responses to gB [7] were also the individuals with evidence of neutralizing antibodies under these experimental conditions. To investigate this possibility the ‘neutralizing activity’ and total gB antibody titre were determined from the previous clinical study [7] and analysed for correlations (Fig. 3). A very simple analysis of the two cohorts where sera were stratified into those with and without evidence of neutralizing antibodies pre-transplant (based on a significant decrease in viral infection in the neutralization assay in Fig. 1a–c) showed that the mean gB antibody titre between the two groups was significantly higher in the individuals we detected with nAbs, although there was a substantial range to the gB IgG titres in both groups (Fig. 3a).

Next we investigated whether we could find any correlation between antibody titre and neutralizing activity specifically in the sera of patients we identified as displayed neutralizing activity. Neutralizing activity (0 % infection=100 % neutralizing activity) was plotted against gB antibody titre (Fig. 3b). Inspection of the plot suggested that with increasing antibody titre there was an increase in neutralizing activity in the sera (Fig. 3b). However, this correlation was non-significant by both Pearson and Spearman rank analyses, although we noted that the *P* value for the Spearman rank analysis was approaching a value of *P*<0.05 (Fig. 3b). Although not conclusive, it does suggest the possibility of a monotonic relationship between titre and neutralizing activity. Thus whilst total gB IgG titre was an important factor, it may not necessarily be the sole determinant of neutralizing activity in the assay.

**Fig. 4.**
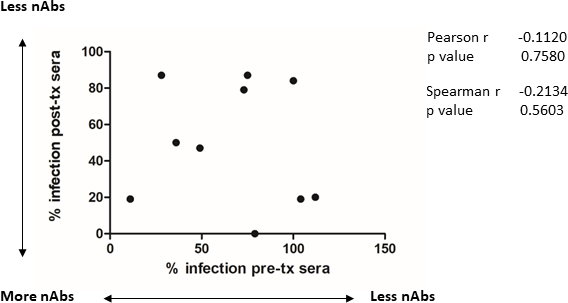
No correlation between detection of neutralizing activity in paired sera from pre-and post-transplant samples. The comparative neutralizing activity of sera of 10 seronegative patients receiving gB/M59 vaccine who proceeded to transplant with a seropositive organ was assessed pre-and post-transplant and subjected to linear regression analysis for correlation.

Previously, we have observed that HCMV-seronegative individuals who were vaccinated prior to transplant displayed a rapid increase in gB antibody titre if the organ received was from an HCMV-seropositive donor [33]. We further observed that this increase in antibody titre also revealed the presence of gB neutralizing antibody responses in post-transplant sera by our standard assays [33]. Thus we next asked whether the individuals with detectable neutralizing antibodies post-transplant were the same individuals in whose sera we detected neutralizing antibodies pre-transplant using our modified cold binding neutralization assay (see Fig. 1). To do this, we analysed the data from the vaccinated R− individuals who received a D+ organ who had been assessed for neutralizing activity in their pre-and post-transplant sera. Ten patients fitted the criteria for the comparison. We plotted the relative neutralizing activity of the pre-transplant (modified assay) and post-transplant (standard assay) sera to test whether there was any correlation. Interestingly, the data show that individuals with evidence of neutralizing activity pre-transplant did not necessarily display the strongest neutralizing antibody response post-transplant (Fig. 4). Consistent with this, there was no direct correlation between the levels of neutralizing activity observed from paired sera taken pre-and post-transplant from the same patient (Fig. 4). For example, three individuals with high levels of neutralizing antibodies post-transplant had no detectable neutralizing antibodies pre-transplant in the cold modified assay.

**Fig. 5.**
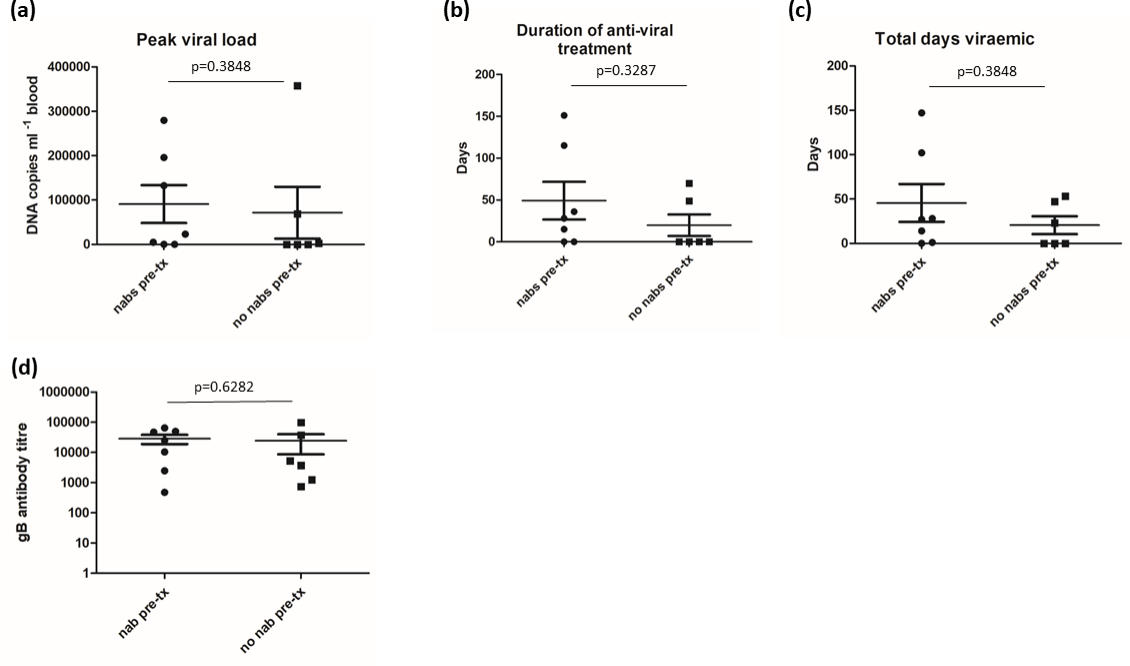
Presence of neutralizing antibodies pre-transplant does not predict better outcomes post-transplant. (a–c) Thirteen HCMV-seronegative individuals who received gB/MF59 vaccine and then proceeded to transplant with an organ from an HCMV-seropositive donor were stratified into those with (nAbs pre-tx) and without (no nAbs pre-tx) and assessed for peak viral load (**a**), duration of anti-viral treatment (**b**) and total days viraemic (**c**). Means were compared using the Mann–Whitney U test assuming a non-parametric distribution. (**d**) The total gB antibody titre in sera taken from 13 HCMV-seronegative patients receiving gB/MF59 vaccine who went on to receive an organ from a HCMV-seropositive donor was calculated for pre-transplant sera defined as possessing neutralizing (nAb pre-tx) or no neutralizing (no nAb pre-tx) antibodies. A comparison of the means was performed using the Mann–Whitney U test assuming a non-parametric distribution.

The utility of these serum samples is that retrospective analyses of parameters measured in the clinical study of the gB vaccine can be performed. Thus we investigated whether the detection of neutralizing antibodies in our modified assay was an indicative correlate of protection post-transplant. To investigate this, patient sera were again stratified into those that did and did not have detectable neutralizing antibodies in our modified assay as described previously (see Fig. 3). We focused our analysis on seronegative individuals who had received the gB vaccine and then received an organ from an HCMV-seropositive donor. In all, 13 patients fitted these criteria – with 7 individuals displaying significant evidence of nAbs pre-transplant and 6 with no significant evidence of nAbs pre-transplant. A tabular analysis showed that 6/7 individuals with detectable nAbs had evidence of viraemia and 3/6 of individuals without nAbs had evidence of viraemia with two requiring anti-viral treatment (see Table 1). An analysis of specific clinical parameters provided no evidence that the presence of nAbs pre-transplant was a predictive correlate of protection (Fig. 5). Specifically, no statistical significant difference in peak viral load, length of anti-viral treatment and duration of viraemia was observed between the two cohorts (Fig. 5a–c). However, we have also observed that gB IgG titre correlated with neutralizing antibody levels (Fig. 3a) and thus a lack of correlation was puzzling given that gB IgG titre was the correlate of protection in the previous phase II study [7]. However, when we analysed the HCMV-seronegative vaccine recipient sera just from patients who received a D+ organ there was no statistical difference between those displaying neutralizing antibodies and those that did not (Fig. 5d), suggesting that the correlation of gB IgG titre with clinical outcome is much stronger than we report for neutralizing antibody activity here.

**Table 1.** Thirteen HCMV-seronegative individuals who received gB/MF59 vaccine and proceeded to transplant with a D+ organ

Patient	nAbs pre-tx	Days viraemia	Peak viral load	Anti-viral treatment (days)
**2**	**N**	**47**	357 470	**49**
**7**	**Y**	**147**	132 585	**151**
**8**	**N**	**53**	69 051	**70**
**5**	**N**	**0**	**0**	**0**
**16**	**Y**	**102**	279 387	**115**
**9**	**N**	**0**	**0**	**0**
**19**	**Y**	**1**	**206**	**0**
**25**	**Y**	**14**	**4952**	**15**
**28**	**Y**	**28**	23 217	**28**
**14**	**N**	**0**	**0**	**0**
**6**	**N**	**23**	**2711**	**0**
**22**	**Y**	**27**	195 774	**36**
**17**	**Y**	**0**	**0**	**0**

**Fig. 6.**
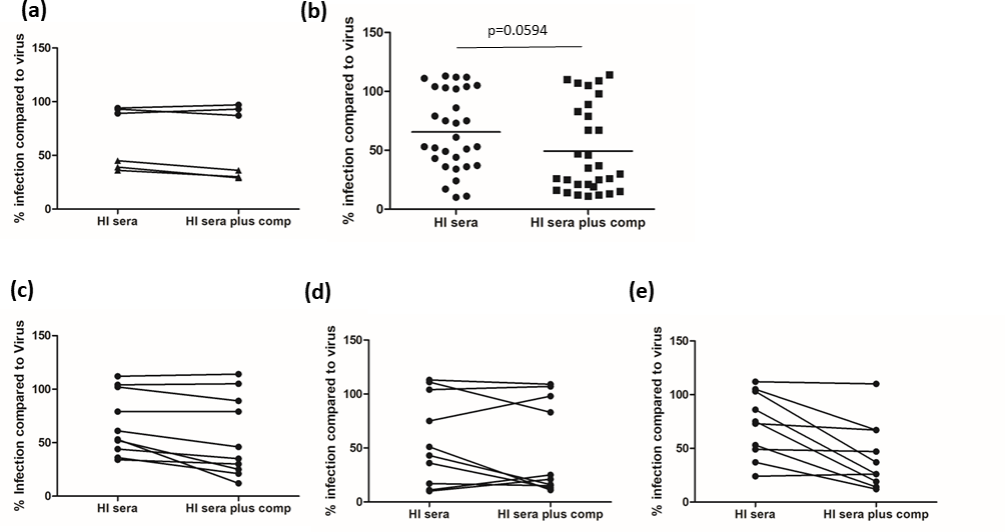
Complement enhances the neutralizing activity of vaccine patient sera in the cold neutralization assay. (a) Sera from healthy HCMV-seronegative (circle) or HCMV-seropositive (triangle) patients were incubated with HFFs previously infected for 1 h at +4 °C in the presence or absence of 5 % guinea pig complement and then assayed for infection by IE IF and quantified by Hermes WiScan. The % infection is expressed relative to a virus-only control (no serum). (**b**) Sera from 30 HCMV-seronegative individuals vaccinated with gB were analysed pre-transplant for neutralizing activity as described in (**a**) with (square) or without (circle) 5 % guinea pig complement. A comparison of the means was performed by Mann–Whitney U test (**b**).**(c–e**) The cumulative data in (**b**) are presented as paired samples of sera showing the impact of complement on the activity of individual patient sera (**c–e**).

These studies thus far had focused on the capacity of antibodies to ‘neutralize’ HCMV infection in the absence of complement. Thus in effect they are acting as blocking antibodies that limit gB activity and function. However, it is well established that the repertoire of gB neutralizing antibody responses incorporates both complement-independent [15] and complement-dependent [38, 39] activities and it has also been demonstrated that complement can enhance the neutralizing activity of antibodies produced in response to gB/MF59 challenge [40]. Thus we tested whether the addition of complement to the assay could enhance any neutralizing activity we observed (Fig. 6). First we confirmed that complement did not specifically promote neutralization with HCMV-seronegative sera and also observed that it had a minor enhancing effect on seropositive sera at the dilution used (Fig. 6a). Next we performed a side-by-side analysis of all 30 heat-inactivated patient sera samples with or without the addition of complement (Fig. 6b–e). The data suggest that the addition of complement increases the general level of neutralizing activity observed but just failed to reach significance (Fig. 6b) when the means of the two conditions were compared. However, when the data were deconvoluted into 10 paired analyses it was clear that complement enhanced some but not all patient sera, which would be consistent with patient sera containing repertoires of complement-dependent and complement-independent neutralizing antibodies(Fig. 6c–e).

## Discussion

Standard neutralization assays (which we and others employ routinely) incubate cell-free HCMV with sera or antibodies and then measure infection via either immuno-staining for viral gene expression or the gold standard plaque reduction assay. In this short report, we demonstrate that inclusion of a +4 °C virus-binding step in the assay has the potential to detect low levels of neutralizing antibodies directed against HCMV. In this case, this was demonstrated using sera obtained from a phase II study of the gB/MF59 vaccine in individuals on the waiting list for organ transplantation [7].

The motivation for the development of this approach was not to replace existing assays – the +4 °C step to promote viral binding is clearly not directly applicable *in vivo* – but driven by our desire to better understand the composition and ontogeny of the immune response in our gB vaccine recipients. Previously, we and others have reported that the gB/MF59 vaccine does not induce readily detectable neutralizing antibody responses in cohorts from two phase II clinical trials [28, 29] and thus concluded that there was no evidence that neutralizing antibodies could be claimed as a correlate of protection. However, following natural infection, gB neutralizing antibodies are clearly made, and so why the vaccine was comparatively poor at inducing neutralizing antibodies remained unclear [26]. A possible explanation is that the mutations introduced into the gB vaccine alter the structure. For example, the transmembrane domain has been deleted (to facilitate secretion for purification) and the furin cleavage site has also been mutated. Furin cleavage is considered to be important for the formation of the native gB trimer [41, 42] and removal likely leads to an increase in the gB monomer in the vaccine [43]. Indeed, work from Liu and colleagues demonstrated that the pre-fusion structure of gB could dramatically change the availability of known antigenic domains of gB when compared with the post-fusion structure of gB [44], which again would be consistent with different humoral responses against the gB vaccine versus native gB.

Despite this, it was still surprising that neutralizing antibodies were largely undetectable in the sera and we remained intrigued that this is not a universal observation: other studies of gB vaccine preparations clearly demonstrate evidence of neutralizing antibody responses directed against other forms of gB [26, 29, 43]. Furthermore, in our own follow-up study of sera taken from *post-transplant* gB vaccine recipients we could also detect evidence of an increase in the gB antibody titre, which was concomitant with the detection of IgG neutralizing antibodies directed against gB as early as 7 days post-HCMV challenge (i.e. 7 days post-organ transplant) [33].

When considering the implications of our later study [33] of the gB antibody response in our post-transplant cohort two immediate explanations for the appearance of detectable neutralizing antibody responses directed against gB could be considered: either challenge with the virus (at the time of transplant with HCMV-positive organ) was boosting a small but pre-existing response generated against the vaccine or *de novo* responses against gB were being formed upon challenge with HCMV. We noted that IgM responses were rarely detected early post-transplant and IgG responses against other HCMV antigens not included in the vaccine developed much later, with no difference between vaccine recipient and placebo controls. Furthermore, the development and maturation of IgG immune responses against HCMV is reported to take much longer than 30 days post-transplant [45, 46]. This led us to hypothesize that the vaccine primes the humoral immune response against gB, which is then rapidly boosted following challenge with HCMV, and that a small component of the initial priming response may include gB neutralizing antibodies, which become detectable in post-transplant sera using our conventional assays due to an increase in titre.

A clear contradiction to our prime–boost hypothesis was our failure to detect neutralizing antibody responses pre-transplant in the same vaccine recipients [28]. However, we hypothesized that if the initial neutralizing antibody response was low it was plausible that a response was made but below the level of detection in our assays *in vitro*. Potentially, neutralizing antibodies were present but not at sufficient concentrations to be effective against high titres of cell-free HCMV used *in vitro*. This led us to investigate alternative strategies to assess for neutralization, including cold neutralization. Using this approach, we identified low but reproducible levels of neutralizing antibodies directed against gB in some, but not all, of our vaccine recipients. The ability to detect neutralizing antibodies in our vaccine sera by this approach prompted a number of potential interpretations.

One interpretation of the data from the vaccine sera was that this method was potentially more sensitive for the detection of neutralizing antibodies. Binding at +4 °C changes the association and dissociation rates of antibody binding, with a decrease in temperature favouring higher avidity antibodies [47]. Thus, potentially the low proportion of neutralizing antibodies in the vaccine sera are high avidity and the +4 °C step favours the binding of these high-avidity antibodies over competing low-avidity antibodies present in the sera. However, we are cautious of this interpretation, as in our original neutralization assays when we incubated HCMV with sera at +4 °C prior to infection no overt evidence of neutralization was detected and thus this likely does not wholly explain the differences [28]. Furthermore, known neutralizing antibodies against gB were much more effective when used at 37 °C compared to +4 °C, suggesting that the protocol itself does not non-specifically enhance neutralizing antibody responses – although of course the assays using purified gB antibodies were not performed in the presence of competing antibodies. That said, we note that it has been demonstrated that a bivalent AD2 antibody (but not an AD4 targeted antibody) can still neutralize HCMV infection post-binding using a similar approach [48]. This appears to be partially in contrast to our data, since we have previously shown that QG1 monoclonal antibodies used in our study are targeted against AD2 [35]. That said, here we demonstrate that our AD2 antibodies are less effective at +4 °C compared to 37 °C, but without detailed side-by-side characterization of different AD2 antibodies it is not sensible to infer too many conclusions apropos the prior study.

A tantalizing alternative explanation for the vaccine sera data presented here is the presence of an antibody response directed against an epitope of gB that is only exposed transiently during the fusion process. The gB trimer undergoes multiple structural changes as it transitions from a pre-fusion to a post-fusion form during the entry process [49], with recent evidence suggesting that gB antibodies directed against AD5 display different activity against different forms [50]. At 37 °C the exposure of these epitopes is possibly more transient if compared to infection at +4 °C, where the virus is stalled at the plasma membrane and thus gB could be in a transitioning state for longer. Thus one possibility is that an epitope is being presented for longer in this assay that is being recognized by neutralizing antibodies in the pre-transplant vaccine sera. If this is coupled with the possibility that the gB/MF59 vaccine presented these antigens more effectively, then it may explain their detection in the sera of vaccine recipients.

It is tempting to speculate that possibly strategies that could increase the concentration of these antibodies may serve to make them functional against HCMV under normal infection conditions – a hypothesis we considered when trying to explain the neutralizing activity of post-transplant vaccine sera where gB antibody titres are increased. That said, the lack of a correlation between the neutralizing activity of pre-and post-transplant sera argues that this is not the explanation here, but instead the neutralizing antibodies detected pre-and post-transplant may be against different regions of gB. Indeed, neutralizing activity in the post-transplant sera was detected by conventional assays. If the cold neutralization approach identifies antibodies with different neutralization profiles, then a lack of correlation, arguably, is unsurprising. Why these responses occur at all is less clear but it may reflect differential presentation of gB epitopes by the vaccine which has been modified [28, 29, 31, 32]. For instance, the presence of gB monomers in the preparation could lead to responses against epitopes hidden in normal virion-associated gB. Additionally, these studies have only been performed in fibroblasts. It could be informative to assess the activity of these neutralizing antibody responses to gB in the context of infection of non-fibroblast cells. Upon infection of epithelial or endothelial cells the presentation of gB epitopes could be different during the process of endocytic entry compared to fibroblasts, where binding and fusion occurs at the plasma membrane. Further characterization of specific responses responsible for these observations could lead to the development of antibodies that can be used to probe differential changes in gB structure during the entry into multiple cell types.

Even though these humoral responses are generated against a vaccine preparation of gB, and thus may not be so common in natural infection, it does not mean that they could not be important. Pathogens are adept at masking epitopes to limit *de novo* immune responses against them or to prevent recognition if they are made [51–53]. However, that does not mean if a humoral (or T cell) response can be generated (e.g. by vaccination) that such responses could be biologically or clinically important. Our work identifying AD6 is direct example of this [32].

From a clinical perspective, we could find no evidence of neutralizing activity in the sera pre-transplant correlating with protection. Indeed, the trend was actually towards poorer clinical outcomes post-transplant – but we highlight a major caveat that this is a retrospective study not powered to formally address this question and so remain cautious in our interpretation of the clinical implications of this. For example, once we stratified for specific parameters, the number of patients eligible for inclusion in the analysis was small. That said, in our previous study of post-transplant sera we did observe a correlation between the detection of neutralizing antibodies post-transplant and better outcomes post-transplant [33] – thus the differential impact as a correlate on outcome of pre-and post-transplant sera neutralizing activity likely explains the lack of a strong correlation between levels of neutralizing activity in paired sera pre-and post-transplant.

In summary, we report data from a modified approach for the characterization of neutralizing antibody responses present at low levels in the sera of gB vaccine recipients and for some patient sera the addition of complement enhanced the neutralizing activity. In doing so, we identify evidence of low levels of neutralizing antibodies in HCMV-seronegative patients who had received the HCMV gB vaccine. The importance of these neutralizing antibody responses is not fully understood, as we observed no correlation between ability to detect neutralizing responses pre-vaccine with better outcomes post-transplant. This is consistent with the increasing evidence that a component of humoral immunity important for the control of HCMV by the gB vaccine includes non-neutralizing antibody effector functions [26, 28, 29, 32, 54, 55]. That said, our drive to present the data from this study is to demonstrate the potential utility of this approach to enrich our understanding of clinical samples and also possibly for the study of HCMV entry and the role of glycoprotein conformation. For example, a panel of antibodies directed against known epitopes within a glycoprotein may show differential neutralizing activity at +4 °C and 37 °C, which could aid the identification of regions that are important for the function of glycoproteins during the different stages of the complex entry process of HCMV.
